# Alveolar hypoxia induces organ‐specific inflammasome‐related inflammation in male mouse lungs

**DOI:** 10.14814/phy2.16143

**Published:** 2024-07-21

**Authors:** Camilla Udjus, Bente Halvorsen, Xiang Yi Kong, Ellen Lund Sagen, Marita Martinsen, Kuan Yang, Else Marit Løberg, Geir Christensen, Ole Henning Skjønsberg, Karl‐Otto Larsen

**Affiliations:** ^1^ Department of Pulmonary Medicine Oslo University Hospital Ullevål and University of Oslo Oslo Norway; ^2^ Institute for Experimental Medical Research Oslo University Hospital Ullevål and University of Oslo Oslo Norway; ^3^ Institute of Clinical Medicine University of Oslo Oslo Norway; ^4^ Research Institute of Internal Medicine Oslo University Hospital Rikshospitalet and University of Oslo Oslo Norway; ^5^ Department of Pathology Oslo University Hospital Ullevål and University of Oslo Oslo Norway; ^6^ K.G. Jebsen Center for Cardiac Research University of Oslo Oslo Norway

**Keywords:** Caspase‐1, hypoxia, IL‐18, inflammation, pulmonary hypertension

## Abstract

Inflammation through activation of caspase‐1, seems to play a role in pulmonary hypertension induced by alveolar hypoxia. Whether alveolar hypoxia induces caspase‐1‐mediated inflammation and influx of leukocytes in other organs than the lungs, is not known. Our aim was to explore sites of caspase‐1‐related inflammation in alveolar hypoxia. Wild type (WT) mice were exposed to environmental hypoxia or room‐air, and organs were analyzed. Right heart catheterization was performed after 14 days of alveolar hypoxia in WT mice and mice transplanted with WT or caspase‐1^−/−^ bone marrow. Hypoxia induced leukocyte accumulation and increased caspase‐1 protein in the lungs, not in other organs. WT mice transplanted with WT or caspase‐1^−/−^ bone marrow showed no difference in pulmonary leukocyte accumulation or development of pulmonary hypertension after alveolar hypoxia. Caspase‐1 and IL‐18 were detected in bronchial epithelium in WT mice, and hypoxia induced IL‐18 secretion from bronchial epithelial cells. IL‐18 stimulation generated IL‐6 mRNA in monocytes. Phosphorylated STAT3 was increased in hypoxic lungs, not in other organs. Alveolar hypoxia induces caspase‐1 activation and leukocyte accumulation specific to the lungs, not in other organs. Caspase‐1 activation and IL‐18 secretion from bronchial epithelial cells might initiate hypoxia‐induced inflammation, leading to pulmonary hypertension.

## 1 INTRODUCTION

A large body of evidence indicates that inflammation plays a role in the development of hypoxic pulmonary hypertension (Cero et al., 2015; Larsen et al., 2008; Pugliese et al., 2015; Rabinovitch et al., 2014; Tuder & Stenmark, 2020; Udjus et al., 2019). In experimental models of hypoxia‐induced pulmonary hypertension, inflammation precedes vascular remodeling, suggesting that inflammation is a cause rather than a consequence of pulmonary hypertension (Cero et al., 2015; Larsen et al., 2008; Pugliese et al., 2015; Rabinovitch et al., 2014; Udjus et al., 2019). Increased levels of proinflammatory cytokines, such as interleukin (IL)‐18, have been shown in patients suffering from chronic lung diseases (Chung, 2001; Imaoka et al., 2008), who are prone to develop both alveolar hypoxia and pulmonary hypertension (Gu et al., 2023). Elevated circulating levels of inflammatory mediators, like IL‐18, have also been observed in pulmonary arterial hypertension (PAH), which seem to correlate with worse clinical outcome (Cracowski et al., 2014; Ross et al., 2012; Soon et al., 2010). IL‐18 is activated by the enzyme caspase‐1, which is the effector molecule of the inflammsomes (Schroder & Tschopp, 2010). The inflammasomes are molecular platforms activated by danger signals, such as infection, leading to engagement of innate immune defenses through activation of IL‐18 and IL‐1β, which can also be activated by non‐canonical pathways including caspase‐8 and ‐11 (Palazon‐Riquelme & Lopez‐Castejon, 2018; Schroder & Tschopp, 2010). In experimental studies, we have previously shown that alveolar hypoxia is sensed as a danger signal, like virus and bacteria, leading to assembly of inflammasome components in the lungs (Cero et al., 2015; Udjus et al., 2019). Caspase‐1 does not influence the important initial event in the development of hypoxic pulmonary hypertension, that is, hypoxic pulmonary vasoconstriction (Swenson, 1985; Udjus et al., 2019). However, alveolar hypoxia activates pulmonary caspase‐1 with maturation of IL‐18 and a corresponding peribronchovascular accumulation of leukocytes, including neutrophils and macrophages, the latter can be due to both recruitment of circulating cells and local proliferation (Cero et al., 2015; Pugliese et al., 2017).

Alveolar hypoxia leads to hypoxemia and in spite of compensatory mechanisms, like opening of additional capillary beds and polycythemia, tissue hypoxia occurs in vital organs (Boero et al., 1985; Cheng et al., 2022; Cole et al., 2016; Shimizu et al., 1989; West, 2017). Persons with alveolar hypoxia due to conditions such as chronic obstructive lung disease (COPD), sleep apnea, or being exposed to high altitude, can present symptoms from several organs like chest pain and dyspnea, proteinuria, tender hepatomegaly and edema, spleen infarction, nausea and diarrhea, and headache and dizziness (Arestegui et al., 2011; Cornwell 3rd et al., 2021; Hackett & Roach, 2001; Henrion et al., 2003; Jefferson et al., 2002; Lane & Githens, 1985; McKenna et al., 2022). Whether involvement of such organs is related to hypoxia‐induced inflammation and triggering of innate immunity through activation of caspase‐1 has not been elucidated. If so, these organs can be potential sources of caspase‐1‐derived inflammatory cytokines during alveolar hypoxia.

In this study, we hypothesized that several organs, in addition to the lungs, are sites of inflammasome activation and inflammatory response induced by alveolar hypoxia. To examine this hypothesis, we exposed mice to a hypoxic environment and harvested the lungs, heart, kidney, liver, spleen, small intestine, and brain to investigate the inflammatory response and activation of caspase‐1. We also wanted to study whether the inflammasome‐driven inflammation induced by hypoxia is related to incoming leukocytes infiltrating the organ, or to resident cells. To elucidate the effect of the inflammasome activation in leukocytes infiltrating the hypoxic organ, we transplanted wild type (WT) mice with caspase‐1 deficient bone marrow and assessed the effect on development of pulmonary hypertension with right heart catheterization.

## 2 RESULTS

### 2.1 Inflammatory response in organs during alveolar hypoxia

**FIGURE 1:**
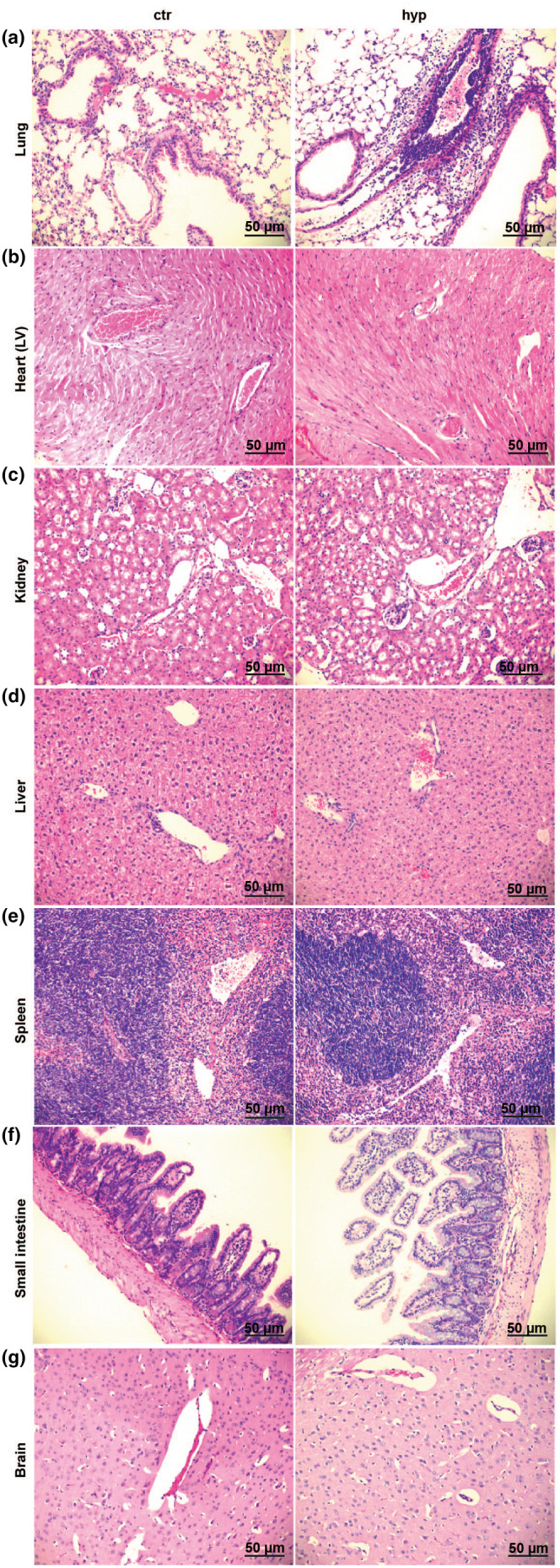
Accumulation of leukocytes in organs from WT mice exposed to 3 days of alveolar hypoxia (hyp) vs. control (ctr). Objective ×20. Scale bar = 50 μm for images. (a) Lung. (b) Heart (left ventricle). (c) Kidney. (d) Liver. (e) Spleen. (f) Small intestine. (g) Brain.

**FIGURE 2:**
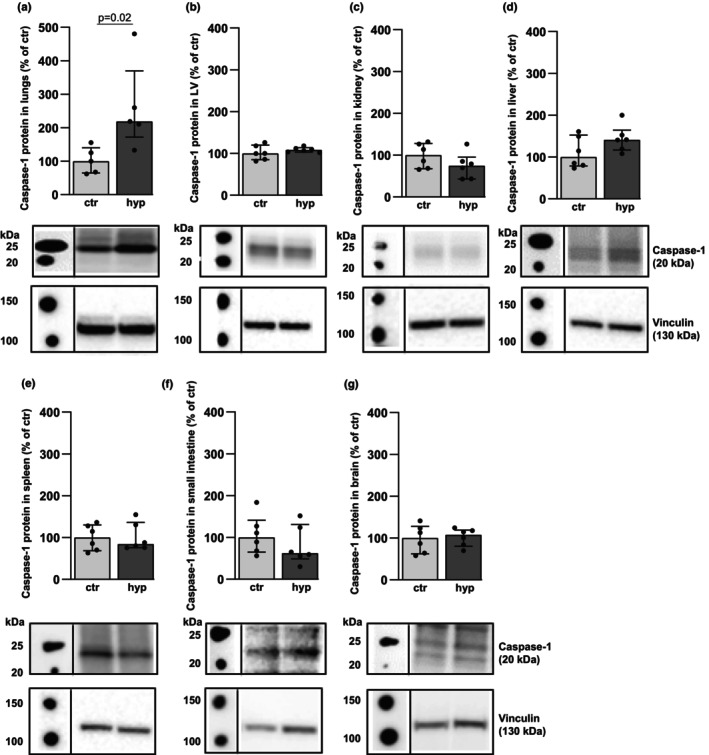
Levels of active caspase‐1 protein in organs from WT mice exposed to 3 days of alveolar hypoxia (hyp) vs. control (ctr). (a) Lung. (b) Heart (left ventricle [LV]). (c) Kidney. (d) Liver. (e) Spleen. (f) Small intestine. (g) Brain. Corresponding Western blots with vinculin as a loading control. Median ± interquartile range (IQR). *n* = 5 or 6 in each group.

A robust perivascular and peribronchial accumulation of leukocytes was observed in the lungs of mice exposed to alveolar hypoxia, compared to mice living in a normoxic environment (control) (Figure 1a) (median histological score 8 [range 6–8] in alveolar hypoxia vs. 0 [range 0] in the control group [*p* < 0.05, *n* = 9/9]). In contrast, no hypoxia‐induced leukocyte accumulation was found in the heart (left ventricle [LV]), kidneys, liver, spleen, small intestine, or in the brain (Figure 1b–g), indicating that the leukocyte accumulation induced by hypoxia seems to be restricted to the organ being directly exposed to low oxygen levels. Activation of inflammasomes, leading to conversion of pro‐caspase to active caspase, is previously shown to elicit influx of leukocytes. Thus, measurement of active caspase‐1, a marker of inflammasome activation, was performed in the lungs and the other organs to study a possible relationship between hypoxia‐induced cellular inflammation and activation of caspase‐1. Increased active caspase‐1 was found in lungs from mice exposed to alveolar hypoxia compared to normoxic controls (Figure 2a). No significant changes in the amounts of active caspase‐1 were found in the LV, kidney, liver, spleen, small intestine, or in the brain in alveolar hypoxia compared to control (Figure 2b–g). Taken together, hypoxia induced increased activation of caspase‐1 in the lungs together with peribrochovascular accumulation of leukocytes, which was not observed in the other organs.

### 2.2 Caspase‐1 in circulating leukocytes does not influence pulmonary inflammation and pulmonary hypertension

**FIGURE 3:**
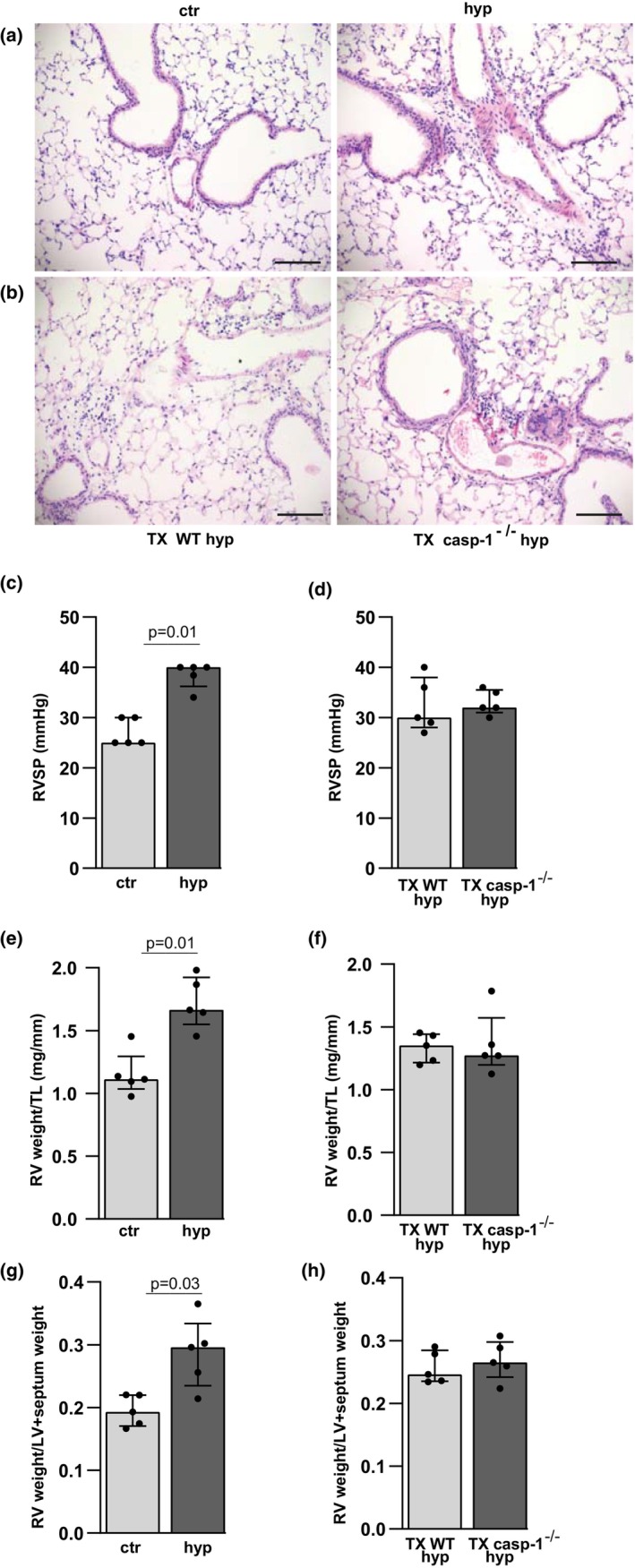
Disruption of caspase‐1 in circulating leukocytes is not protective against hypoxia‐induced accumulation of leukocytes and pulmonary hypertension. Mice were exposed to chronic alveolar hypoxia (hyp) or room air (control [ctr]). (a) Images from lungs of WT ctr and WT hyp. (b) Images from lungs of WT mice transplanted with WT bone marrow (TX WT hyp) or transplanted with caspase‐1^−/−^ bone marrow (TX casp‐1^−/−^ hyp). Objective ×20 in a and b. Scale bar = 50 μm for images. (c) Right ventricular systolic pressure (RVSP) in WT ctr vs. WT hyp. (d) RVSP in TX WT hyp vs. TX casp‐1^−/−^ hyp. (e) RV weight normalized to TL (RV weight) in WT ctr vs.WT hyp. (f) RV weight/TL in TX WT hyp vs. TX casp‐1^−/−^ hyp. (g) RV weight normalized to weight of LV plus septum (RV weight/LV + septum weight) in WT ctr vs.WT hyp. (h) RV weight/LV + septum weight in TX WT hyp vs. TX casp‐1^−/−^ hyp. *n* = 5 in each group. Median ± interquartile range (IQR).

After having identified the lungs as a target organ for hypoxia‐induced cellular inflammation and caspase‐1 activation, we examined whether the presence of the inflammasome effector caspase‐1 in circulating leukocytes was crucial for the development of pulmonary inflammation and pulmonary hypertension in chronic hypoxia. First, accumulation of leukocytes in the lungs was observed in WT mice exposed to chronic hypoxia compared to normoxic controls (Figure 3a). Chronic hypoxia also induced pulmonary leukocyte accumulations in mice transplanted with caspase‐1^−/−^ bone marrow and in mice transplanted with WT bone marrow (Figure 3b), indicating that hypoxia‐induced cellular accumulation in the lungs was not dependent on inflammasome activity in the incoming leukocytes. Since inflammation is involved in the development of pulmonary hypertension, we next explored the effect of caspase‐1 disruption in circulating leukocytes on the development of hypoxia‐induced pulmonary hypertension. Increased right ventricular systolic pressure (RVSP), right ventricular (RV) weight/tibial length (TL) ratio, and RV/LV + septum ratio were found in WT mice exposed to chronic hypoxia, confirming development of pulmonary hypertension, compared to normoxic controls (Figure 3c,e,g). RVSP was not significantly changed in WT mice with caspase‐1^−/−^ bone marrow compared to WT mice with WT bone marrow after chronic hypoxia (Figure 3d). This finding was supported by unaltered RV weight/TL ratio (Figure 3f) and RV/LV + septum ratio (Figure 3h) in hypoxic WT mice with caspase‐1^−/−^ bone marrow compared to hypoxic WT mice with WT bone marrow, indicating that the previously observed attenuation of hypoxia‐induced pulmonary hypertension in ordinary caspase‐1^−/−^ mice (Udjus et al., 2019) is not caused by inflammasome activation in leukocytes infiltrating the lungs.

### 2.3 Caspase‐1 and IL‐18 in resident pulmonary cells induce IL‐6 generation

**FIGURE 4:**
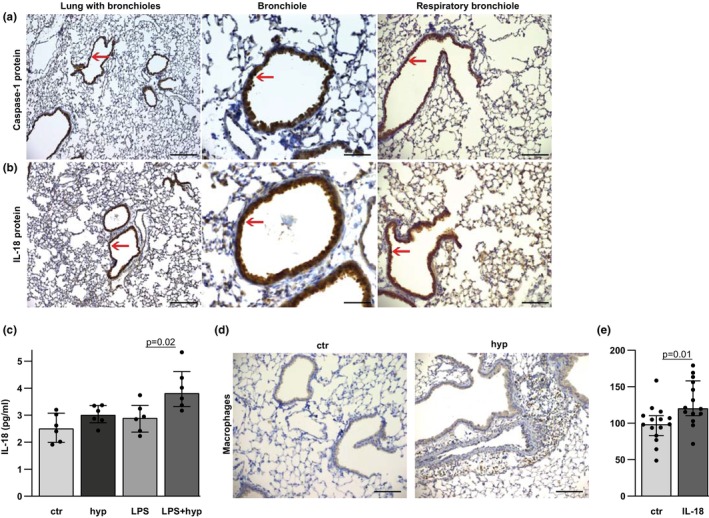
Caspase‐1 and interleukin (IL)‐18 in bronchial epithelium in the lungs, and in vitro hypoxia induces secretion of IL‐18 from primed BEAS‐2B bronchial epithelial cells. (a) Representative images of active caspase‐1 (p20) immunostaining in WT mice. Positive caspase‐1 staining in bronchial epithelium is indicated by red arrow. (b) IL‐18 immunostaining. Positive IL‐18 staining in bronchial epithelium is indicated by red arrow. Lung with bronchioles; objective ×10 and scale bar = 100 μm. Bronchiole; objective ×40 and scale bar = 25 μm. Respiratory bronchiole; objective ×20 and scale bar = 50 μm. (c) Concentrations of IL‐18 in media from BEAS‐2B cells in normoxia (ctr), 1% oxygen (hyp), priming with LPS (LPS) or priming with LPS and 1% oxygen (LPS + hyp). *n* = 6 in each group. (d) Representative images of peribronchovascular accumulations of F4/80^+^ monocytes/macrophages in WT mice exposed to 3 days of alveolar hypoxia (hyp) vs. control (ctr). Objective ×20. Scale bar = 50 μm for images. (e) IL‐6 mRNA levels normalized to β‐Actin mRNA in monocytes stimulated with IL‐18 50 ng/mL for 6 h vs. unstimulated cells (control [ctr]). *n* = 14 or 15 in each group. Median ± interquartile range (IQR).

Knowing that caspase‐1 in incoming leukocytes does not seem pivotal for cellular pulmonary inflammation and hypoxia‐induced pulmonary hypertension, we next examined whether resident cells in the lungs contain caspase‐1 and thus have the ability to induce hypoxia‐induced inflammation and pulmonary hypertension. A positive caspase‐1 staining in bronchial epithelial cells to the level of respiratory bronchioles was observed by immunohistochemical analysis in lungs from WT mice, showing that the effector of the inflammasome is present in cells being directly exposed to a hypoxic environment (Figure 4a). Similarly, positive IL‐18 staining was found in bronchial epithelial cells in lungs from WT mice, and not in the alveolar epithelial cells (Figure 4b), indicating that bronchial epithelial cells to the level of respiratory bronchioles can be sources of IL‐18 upon activation by caspase‐1. We then examined whether hypoxic stimulation of BEAS‐2B bronchial epithelial cells could lead to release of IL‐18. Hypoxia‐stimulated BEAS‐2B cells, primed with LPS, secreted increased levels of IL‐18 in the surrounding medium, while no significant increase in IL‐18 concentrations were observed after stimulation with moderate hypoxia or LPS alone (Figure 4c). In vivo, alveolar hypoxia induced peribronchovascular accumulation of F4/80^+^ macrophages/monocytes in WT mice (Figure 4d), which could be exposed to IL‐18 from bronchial epithelium. In vitro, IL‐18 stimulation induced generation of IL‐6 mRNA in monocytes (Figure 4e). Thus, hypoxia can activate caspase‐1 leading to release of IL‐18 from bronchial epithelial cells generating IL‐6 expression in adjacent monocytes/macrophages.

### 2.4 Alveolar hypoxia induces STAT3 phosphorylation in the lungs

**FIGURE 5:**
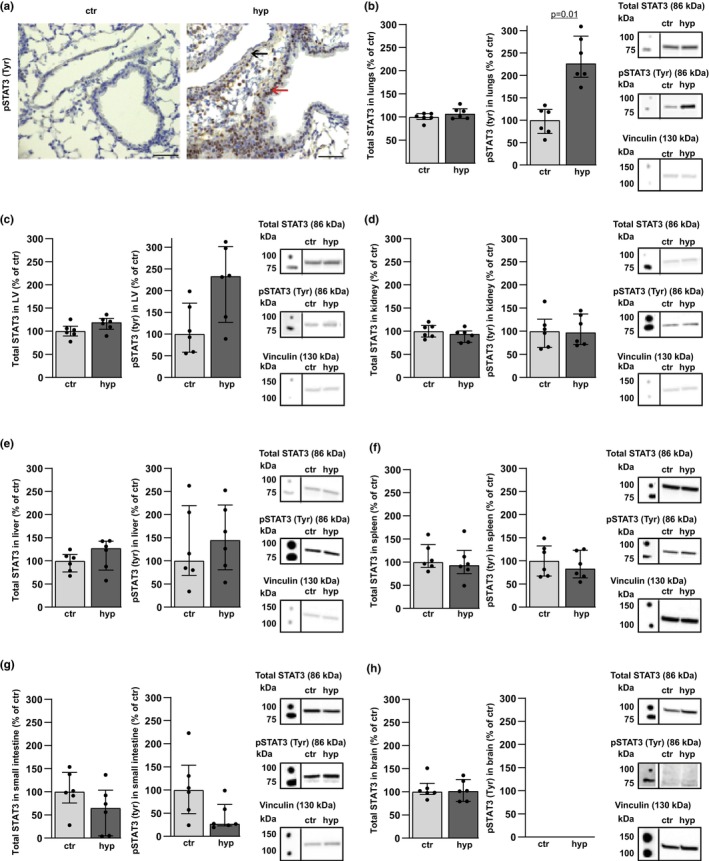
Hypoxia induces phosphorylated (p) STAT3 (Tyr705) in the lungs. Pulmonary pSTAT3 (Tyr705) immunostaining and levels of total STAT3 and pSTAT3 (Tyr705) proteins in organs from WT mice exposed to 3 days of alveolar hypoxia (hyp) vs. control (ctr). (a) Representative images of pSTAT3 (Tyr705) immunostaining in lung with longitudinal section of pulmonary vessel (black arrow) and epithelium in adjacent bronchus (red arrow) after chronic alveolar hypoxia. Objective ×40. Scale bar = 25 μm for images. (b) Total STAT3 and pSTAT3 (Tyr705) protein levels in lung. (c) Heart (left ventricle [LV]). (d) Kidney. (e) Liver. (f) Spleen. (g) Small intestine. (h) Brain. Corresponding Western blots with vinculin as a loading control. *n* = 6 in each group. Median ± interquartile range (IQR).

IL‐6 signals via STAT3 and knowing that STAT3 signaling is linked to both pulmonary hypertension and inflammation, we studied whether total STAT3 and Tyr705 phosphorylation were increased in the investigated organs during hypoxia. Positive pSTAT3 (Tyr705) immunostaining was observed in the wall of medium‐sized and small pulmonary vessels and in the epithelium of adjacent bronchi from WT mice after chronic hypoxia compared to control (Figure 5a). Hypoxia did not alter the level of total STAT3 protein in the lungs, whereas the amount of pulmonary pSTAT3 (Tyr705) protein was increased in alveolar hypoxia compared to control (Figure 5b). No significant changes in the amounts of total STAT3 or pSTAT3 (Tyr705) proteins were found in the other organs in alveolar hypoxia compared to control (Figure 5c–h). Thus, in alveolar hypoxia, elevated levels of pulmonary phosphorylated STAT3 were detected in the smooth muscle cell (SMC) layer in pulmonary vessels and in bronchial epithelium, whereas phosphorylated STAT3 protein levels were unaltered in other organs.

## 3 DISCUSSION

Sources of hypoxia‐induced inflammation related to caspase‐1 were investigated, and increased caspase‐1 protein and perivascular leukocyte accumulation were observed in the lungs in alveolar hypoxia. Previous studies have documented that alveolar hypoxia induces both systemic hypoxaemia and tissue hypoxia in the organs studied in this investigation (Bishop et al., 2013; Boero et al., 1985; Cheng et al., 2022; Cole et al., 2016; Shimizu et al., 1989; West, 2017). We were unable to detect any significant changes in caspase‐1 protein levels or influx of leukocytes in the heart, kidney, liver, spleen, small intestine, or brain indicating that these organs do not seem to be sources of caspase‐1‐related inflammation during hypoxia. Thus, clinical manifestations related to alveolar hypoxia in other organs (Kent et al., 2011) might be triggered by circulating inflammatory cytokines generated in the lungs (Barnes & Celli, 2009; Larsen et al., 2008), and not local activation of innate immunity. This is consistent with increased levels of circulating IL‐18 both in experimental alveolar hypoxia (Larsen et al., 2008) and in patients with hypoxic lung diseases (Imaoka et al., 2008). Pulmonary inflammation in mice could theoretically be related to altered ventilatory pattern during exposure to environmental hypoxia. However, mice depleted of caspase‐1 do not develop pulmonary inflammation, as observed in their WT littermates with identical genetic background (C57Bl6) living in the same hypoxic environment (Udjus et al., 2019). This makes it less likely that an altered ventilatory pattern is responsible for the hypoxia‐induced pulmonary inflammation with peribronchovasular accumulation of leukocytes. Since hypoxic caspase‐1 deficient mice develop a similar degree of hypoxic pulmonary vasoconstriction as WT mice, without showing accumulation of leukocytes in the lungs (Udjus et al., 2019), it also seems unlikely that the peribronchovascular accumulation of leukocytes is caused by hydrostatic pulmonary edema due to pulmonary vasoconstriction. Since both others and our previous studies have indicated that pulmonary inflammation has a central role in the development of hypoxia‐induced pulmonary hypertension (Frid et al., 2006), we examined the impact of caspase‐1 in circulating leukocytes in this process. Mice transplanted with caspase‐1^−/−^ or WT bone marrow developed a similar accumulation of leukocytes in the lungs and similar levels of pulmonary hypertension in alveolar hypoxia, indicating that the inflammasome and caspase‐1 activation in incoming leukocytes are not prerequisites for development of pulmonary hypertension. Thus, our attention was drawn to inflammation induced by caspase‐1 in resident cells in the lungs.

In the lungs, we observed positive immunostaining of the inflammasome effector caspase‐1 in the bronchial epithelium. The epithelial lining of both the lungs and the gut has been regarded as a protective barrier against pathogens and physical and chemical injury. As the primary organ sensing the external environment, it is apparent that the epithelium is a vital sensor of environmental cues (Palazon‐Riquelme & Lopez‐Castejon, 2018; Roan et al., 2019). The inflammasome is of importance in this context, and upon sensing damage‐ and pathogen‐associated molecular patterns (DAMPs and PAMPs), the epithelium is able to secrete immune mediators, such as the inflammasome‐regulated IL‐18 (Kaur et al., 2021). In human biopsies, IL‐18 protein was observed in the airway epithelium of healthy control individuals (Cameron et al., 1999), which is in concordance with our finding of positive IL‐18 immunostaining in the epithelium of lungs from normoxic control mice. Accordingly, IL‐18 is present in basal normoxic conditions, available to be released when the inflammasome in the epithelium senses infectious or sterile inflammatory stimuli and activates its effector caspase‐1. Morisawa and coworkers showed a role for IL‐18 in the development of pulmonary hypertension related to lung disease and hypoxia by attenuated hypoxic pulmonary hypertension in mice with IL‐18 disruption (Morisawa et al., 2016), and in transgenic mice that overproduced mature IL‐18, Hoshino et al. found accumulation of pulmonary leukocytes and pulmonary hypertension, in addition to emphysema (Hoshino et al., 2007). In other organs, IL‐18 overproduction led to cardiomegaly due to dilatation of a hypertrophied right ventricle, without signs of inflammation in the heart, kidney, liver, intestine or brain, and moderate splenomegali (Hoshino et al., 2007), showing similar organ engagement in IL‐18 overproduction as in our study of alveolar hypoxia. In the current study, IL‐18 was localized to the epithelium, and hypoxia induced secretion of IL‐18 from primed bronchial epithelial cells in vitro. IL‐18 can be released through pores at the basal part of the epithelial cells, thereby influencing adjacent cells (Palazon‐Riquelme & Lopez‐Castejon, 2018), including the observed accumulations of leukocytes with macrophages/monocytes in alveolar hypoxia.

Cellular crosstalk among epithelial cells and other cell types, such as macrophages, takes place in the development of the respiratory system and later in chronic lung disorders (Zepp & Morrisey, 2019). Likewise, cellular interactions have been shown in hypoxic pulmonary hypertension, for instance between resident macrophages and client cells such as fibroblasts or SMCs, inducing cytokines and growth factors leading to SMC proliferation, which is crucial in the development of pulmonary hypertension (Pugliese et al., 2015). In this study of hypoxic pulmonary hypertension, epithelial derived IL‐18 can be a trigger of pulmonary inflammation and pulmonary hypertension via IL‐6 generation in infiltrating monocytes/macrophages and STAT3 signaling. Increased levels of pSTAT3 (Tyr705) were found in SMCs in PAH (Courboulin et al., 2011), and in alveolar hypoxia we found increased pulmonary level of pSTAT3 (Tyr705) protein localized to the muscle layer in pulmonary vessels by immunohistochemistry, indicating STAT3 as a mechanism of SMC proliferation in hypoxic pulmonary hypertension. Notably, STAT3 may also mediate inflammation, and in alveolar hypoxia a positive pSTAT3 (Tyr705) immunostaining, observed in the bronchial epithelium, is able to facilitate accumulation of leukocytes in the lungs (Nikolskii et al., 2021). In the heart, kidney, liver, spleen, small intestine, and brain, the levels of total STAT3 and pSTAT3 (Tyr705) proteins were not altered in alveolar hypoxia compared to respective controls, being consistent with lack of leukocyte accumulation in these organs, hence supporting STAT3 as a mediator of inflammation in alveolar hypoxia. In the lungs, the perivascular accumulation of leukocytes correlates directly with pulmonary vascular remodeling in hypoxia (Pugliese et al., 2015). Accordingly, our findings of hypoxia‐induced pSTAT3 (Tyr705) protein in the epithelium and in the smooth muscle layer of pulmonary vessels are consistent with previous findings of STAT3 as both a facilitator of inflammation and a mediator in vascular SMC proliferation (Bisserier et al., 2021; Courboulin et al., 2011; Nikolskii et al., 2021).

## 4 MATERIALS AND METHODS

### 4.1 Mice

All our investigations were approved by the Norwegian Animal Research Authority (ID 19464) and conform to the Guide for the Care and Use of Laboratory Animals (NIH Publication, 8th Edition, 2011). Caspase‐1^−/−^ mice, caspase‐1/11 knock‐out mice backcrossed 10 generations with C57BL/6 background (Kayagaki et al., 2011; Wang et al., 1998), and WT littermates were utilized. WT C57Black/6 J mice were obtained from Janvier Labs (Saint Berthevin Cedex, France). Caspase‐1^−/−^ mice (B6N.129S2‐Casp1tm1Flv/Js) were acquired from The Jackson Laboratory (Bar Harbor, ME). Genotyping of caspase‐1^−/−^ and WT littermates is continuously performed at our institute (IEMR, Oslo University Hospital, Oslo, Norway). Backcrossing to C57Black/6 J, by new heterozygote breeding and subsequent homozygote breeding, is carried out to prevent genetic drift of caspase‐1^−/−^ and their littermates. A total number of 87 mice were used in the study.

### 4.2 Experimental protocol

Eight‐week‐old male WT mice were in open‐top animal cages covered by wire bar lid and either exposed to 10% oxygen in a normobaric, sealed chamber (hypoxia) where the oxygen level was kept constant by the ProOx 110 oxygen controller (BioSpherix, Redfield, NY), or normoxic air (control) for 3 or 14 days (Larsen et al., 2006). The hypoxia chamber was opened once a week to replace used animal cages with clean ones. The barometric pressure was 100.40 kPa with daily fluctuations of up to 0.31 kPa. The concentration of carbon dioxide, measured by a Beckman LB‐2 Medical Gas Analyzer for carbon dioxide (Beckman Coulter, Indianapolis, IN), was kept <0.4% inside the chamber and the humidity in the chamber was 55 ± 10%. Inhalation of isoflurane 2.5%–5% was used as anesthesia during invasive procedures performed at an operation table outside the hypoxia chamber. Lungs, heart (LV), kidney, liver, spleen, small intestine, and brain were rapidly excised and immediately snap‐frozen in liquid nitrogen and stored at −80°C. The atria were quickly removed from the heart, and the RV was separated from the LV before freezing.

### 4.3 Bone marrow chimeras

Bone marrow recipient mice (eight‐week‐old WT females) were irradiated with two doses of 6 Gray with 4 h rest between each radiation on day 0. The next day (day 1) bone marrow was isolated from femurs from male caspase‐1 deficient and WT littermate mice (eight‐week‐old). Briefly, the bones were rinsed with 70% ethanol and ice‐cold PBS under sterile conditions. The epiphyses of each bone were cut, bone marrow cells were flushed with 10 mL ice‐cold PBS and filtered through a 40 μm mesh. The irradiated mice received 1.0 × 10^6^ bone marrow cells from the donor mice through tail vein injections on day 1, and acidic (pH 2.5) sterile water was provided ad libitum until termination to prevent infections. To verify adequate engraftment of new bone marrow, blood samples were collected from the calf vein to isolate DNA from peripheral blood mononuclear cells (PBMC), and analyzed Y‐chromosome linked genes by PCR (An & Kang, 2013) (Figure S1). Individuals with successfully engrafted donor cells were further used in experiments. After 8 weeks of recovery, transplanted mice were placed in hypoxic conditions 9 weeks after bone marrow transplantation together with WT mice.

### 4.4 Assessment of RVSP

After 14 days of chronic hypoxic exposure (10% oxygen) (Larsen et al., 2006), RV characterization was performed to evaluate the effect of caspase‐1 depletion in leukocytes on the degree of pulmonary hypertension. Using a micro pressure transducer (Samba Preclin 420 LP transducer, Samba Sensors, Västra Frölunda, Sweden), inserted into the RV via the right internal jugular vein, RVSP was measured. Diadem software (National Instruments, Austin, TX) was used to analyze the data.

### 4.5 Western blotting

Frozen organs (lungs, heart, kidney, liver, spleen, small intestine and brain) from WT mice, subjected to 3 days of hypoxia or normoxia, were homogenized (Polytron 1200 homogenizer) in tissue protein extraction reagent (T‐PER, #78510, Thermo Fisher Scientific, Waltham, MA). Frozen organ tissues were disrupted (TissueLyser II) in PBS buffer containing 1% Triton and 0.1% Tween. Protease and phosphatase inhibitor cocktail tablets (#05056489001, #04906837001, Roche Diagnostics, Germany) were added to the solution. Protein concentrations from organ supernatants were quantified using micro‐BCA protein assay kit (#23235, Thermo Fisher Scientific). Sample buffer containing 50% sucrose, 7.5% SDS, 62.5 mM Tris–HCl (pH 6.8), 2 mM EDTA (pH 7.5), 3.1% DTT, and 0.01% bromophenol blue was added to the protein extracts. After heating the sample at 94°C for 5 min, proteins were size fractionated on a Criterion Precast Gel (4–15% Tris–HCl, 18 W) and Midi Format (0.2 μm) PVDF membranes, single application (Bio‐Rad Laboratories, Oslo, Norway). Blots were blocked in 3% BSA in TBST (tris‐buffered saline with 1% Tween) and incubated with primary (1:500) overnight and secondary (1:2000) antibodies for 1 h in room temperature. Blots were developed using Amersham ECL Prime (GE HealthCare, UK) and visualized in LAS‐4000 (Fujifilm, Japan). For quantification of the band intensity, Image Quant TL software (Amersham Biosciences, UK) was used. The primary antibodies used were anti‐caspase‐1 (#14–9832‐80, Thermo Fisher Scientific, Waltham, MA), phospho (p)‐Stat3 (Tyr705) (D3A7) (no. 9145) and Stat3 (124H6) (no. 9139) both from Cell Signaling Technology (Danvers, MA). Anti‐vinculin (#V9131, Sigma‐Aldrich, St. Louis, MO) was used as loading control. As secondary antibody, goat IgG HRP‐conjugated antibody (#HAF109, Healthcare, UK) was used.

### 4.6 Histology

Lungs, heart, kidney, liver, spleen, small intestine, and brain from WT normoxic and hypoxic mice (*n* = 3) and lungs from transplanted mice (*n* = 5) in hypoxia were fixed in 4% paraformaldehyde. Tissue blocks (0.5 cm thick) from each organ were embedded in paraffin and sliced in 3 μm thick sections. Lungs were first inflated by intratracheal installation of 4% paraformaldehyde with a constant pressure of 25 cm H_2_O (Fujita et al., 2004). The sections from the organs were stained with hematoxylin (#01800) and eosin (#01650 both from Histolab Products AS, Västra Frölunda, Sweden). Peribronchovascular pulmonary inflammation in nine fields from three normoxic and hypoxic mice was scored from 0 (normal) to a maximum score of 8 (Koltsida et al., 2013; Xirakia et al., 2010).

### 4.7 Immunohistochemistry

Formalin‐fixed paraffin‐embedded 3‐μm sections were deparaffinized, rehydrated and demasked in microwave oven for 24 min in Target Retrieval Solution (TRS) pH 6.00–6.2. The sections were counterstained with hematoxylin. Antibodies against caspase‐1 (#ab138483, titer 1:800), IL‐18 (#ab71495, titer 1:100), both from Abcam, Cambridge, UK, F4/80 (no. 14–4801, titer 1:50, eBioscience, San Diego, CA), and pStat3 (Tyr705) (no. 9145, titer 1:50, Cell Signaling Technology) were utilized. The antigen–antibody reaction was visualized with DAKO EnVision horseradish peroxidase system (DAKO Cytomation Norden, Glostrup, Denmark) with 3.3′‐ diaminobenzidine as the chromogen.

### 4.8 In vitro experiments

BEAS‐2B bronchial epithelial cells (ATCC, VA) were grown in plates pre‐coated with bronchial epithelial cell basal medium (BEBM) with additives (Lonza/Clonetics, Basel, Switzerland) according to the manufacturer's instruction. The cells were incubated with medium or medium containing 10 ng/mL lipopolysaccharide (LPS) (#L2630, Sigma Aldrich, MO) and either incubated in normoxia with 5% CO_2_ or in a New Brunswick hypoxia chamber (Eppendorf AG, Hauppauge, NY) with 1% O_2_ and 5% CO_2_ for 6 h as previously described (Udjus et al., 2019). Conditioned media (CM) were quantified for IL‐18 protein using a U‐plex Biomarker Group 1 assay kit (#K151VJK) from Mesoscale Discovery (Rockville, MD) utilizing a QuickPlex SQ120 (#K15067L‐1). To explore a possible interaction between IL‐18 secretion and infiltrating monocytes/macrophages, human PBMC were isolated from buffy coats of healthy donors (Blodbanken, Oslo University Hospital, Oslo, Norway) by Lymphoprep (Aleris Technology AS, Oslo, Norway), gradient centrifuged, washed with serum‐free RPMI 1640 (Biowest, Bradenton, FL), and incubated in 12‐well plates (3 × 10^6^ cells/well) in RPMI overnight. After incubation, plastic adhered primary monocytes were washed once with RPMI 1640 before stimulation with recombinant human IL‐18, 50 ng/mL (#9124‐IL, R&D Systems, Minneapolis, MN) vs. unstimulated cells (control) in RPMI for 6 h. The cells were washed once with ice‐cold PBS and lysated in Buffer RLT (Qiagen, Hilden, Germany) for analysis of gene expression. Total RNA was isolated using RNeasy Mini Kit in a QIAcube Connect automated isolation machine (Qiagen). Concentrations of total RNA was quantified on DeNovix Spectrophotometer and reverse transcription was made from 750 ng total RNA with Quanta qScript cDNA Synthesis Kit (Quantabio, Beverly, MA). Quantification of IL‐6 mRNA was performed using Perfecta SYBR Green Supermix dye (#95054‐02 K, Quantabio, Beverly, MA) in RT‐qPCR (Biorad CFX 384, Biorad Laboratories, Hercules, CA) and normalized to β‐actin mRNA. The relative level was calculated by relative quantification (2^−ΔΔCt^) method.

### 4.9 Statistical analysis

Data are presented as median ± interquartile range (IQR). To compare two groups, Mann–Whitney rank‐sum test was applied. For multiple comparisons, Tukey's multiple comparisons test was performed. *p*‐values <0.05 was considered statistically significant.

## 5 CONCLUSION

In this experimental study of mice with alveolar hypoxia, the lung seems to be the main organ for hypoxia‐induced inflammation through local activation of innate immunity. In the search for sources of hypoxia‐induced inflammation, we found that airway epithelium, which is the first to be exposed to a hypoxic environment, has abilities to trigger caspase‐1‐related inflammation in the lungs through activation of caspase‐1 and the inflammatory mediator IL‐18. A similar process was not observed in other organs, indicating that the lung is the main source of circulating IL‐18 during hypoxia. Bronchial epithelial cells exposed to hypoxia secrete IL‐18 which is able to induce IL‐6 mRNA in monocytes. Hypoxia‐induced STAT3 phosphorylation in the bronchial epithelium can facilitate inflammation, and pSTAT3 (Tyr705) expression in pulmonary vessels may mediate proliferation of SMCs, leading to hypoxic pulmonary hypertension.

## FUNDING INFORMATION

Dr. Udjus is employed by the Institute of Clinical Medisin, University of Oslo, Oslo, and no additional funding was received.

## CONFLICT OF INTEREST STATEMENT

The authors report there are no competing interests to declare.

## ETHICS STATEMENT

The study was approved by the Norwegian Animal Research Authority (ID 19464) and conforms to the Guide for the Care and Use of Laboratory Animals (NIH Publication, 8th Edition, 2011).

## Supporting information


Figure S1.

## Data Availability

Data are available on request.
